# Debonding Detection in Hidden Frame Supported Glass Curtain Walls Using the Nonlinear Ultrasonic Modulation Method with Piezoceramic Transducers

**DOI:** 10.3390/s18072094

**Published:** 2018-06-29

**Authors:** Xiaobin Hong, Yuan Liu, Yonghong Liufu, Peisong Lin

**Affiliations:** 1 School of Mechanical & Automotive Engineering, South China University of Technology, Guangzhou 510641, China; mexbhong@scut.edu.cn (X.H.); 15270833143@163.com (Y.L.); Lnpsong@163.com (P.L.); 2 School of Physics and Optoelectronics, South China University of Technology, Guangzhou 510641, China

**Keywords:** hidden frame supported glass curtain wall, nonlinear ultrasonic modulation, debonding, empirical mode decomposition

## Abstract

Debonding defects are common and they are the main reason for the failure of hidden frame supported glass curtain walls, which are widely used as an external enclosure and decorative structure. In this paper, a debonding detection method for hidden frame supported glass curtain walls is developed based on nonlinear ultrasonic modulation and piezoceramic transducers. First, the excitation frequency was determined according to the response characteristics. Then, empirical mode decomposition (EMD) was applied to extract the feature components. After discrete Fourier transform (DFT), the nonlinear coefficients were calculated to evaluate the debonding defect. Finally, the experimental setup was established and a series of experiments were carried out to verify the feasibility and effectiveness of the nonlinear ultrasonic modulation method. The nonlinear harmonics detection method was also investigated and it was compared with the nonlinear ultrasonic modulation method. The detection effect at different temperatures and impact were studied. The results showed that the nonlinear coefficient increases with the debonding length. The mean squared error (MSE) of the nonlinear ultrasonic modulation method was improved by 41% compared with the nonlinear harmonics method. The nonlinear ultrasonic modulation method can successfully detect debonding defects in hidden frame supported glass curtain walls at different temperatures and impact.

## 1. Introduction

The hidden frame supported glass curtain wall is widely used in the construction field due to its dexterity, lightness, transparency and structural beauty. It provides all the required functions of an external enclosure and decorative structure, which usually do not contribute to the load-bearing ability. The glass plate of the hidden frame supported glass curtain wall is directly bonded to the aluminum alloy subframe by silicone structural adhesive. With the increase in service life, problems with the quality of the hidden frame supported glass curtain wall have attracted significant attention. Because of the effects of long-term thermal stress and environmental aging, hidden frame supported glass curtain walls inevitably suffer from defects such as cracks, bubbles, lamination and debonding defects. In particular, debonding defects are very common in hidden frame supported glass curtain walls. Once there is a debonding defect, it may cause the curtain wall glass to fall off, which may lead to accidents and heavy property losses. Therefore, there is a great need to develop an effective method for debonding detection in hidden frame supported glass curtain walls. 

Various methods have been proposed for debonding detection in hidden frame supported glass curtain walls. The commonly used detection methods are suction cup and balloon methods which apply exerted pressure on the glass by a suction cup or balloon to simulate wind load. The stress and displacement are used to evaluate the quality of the glass curtain wall [1,2]. However, before testing, the balloon or mechanical devices must by installed to apply the appropriate load which leads to low efficiency [2,3]. Compared with the suction cup and balloon method, vibration testing methods have greatly improved the detection process. For example, Chen et al. [4] detected and evaluated debonding defects using an FFT power spectrum method. The contribution of the power spectrum peak to the total power was used as an evaluation indicator of the length of the debonding. Gu et al. [5] developed a dynamic identification method for hidden frame supported glass curtain walls, and the vibration transmissibility was used as a damage identification index based on the Hilbert-Huang transform (HHT). Nevertheless, it is necessary to impose an external impact on the glass curtain wall using the vibration testing method. Once the glass curtain wall has been defective, the glass curtain wall is likely to fall off during the detection process.

At the same time, various ultrasonic testing methods have been proposed for detecting debonding defects, such as ultrasonic scanning and ultrasonic guided waves. For example, Liu et al. [6] detected the debonding defects in thermal barrier coatings based on ultrasonic scanning technology, and the amplitude and phase information were used to evaluate the debonding defect. Pärnänen et al. [7] evaluated the debonding and impact damage by ultrasonic scanning to investigate the relationship between debonding defects and the metal’s surface morphology. Park et al. [8] established a noncontact test system through laser ultrasonic scanning technology to test debonding defects in composite structures. However, the ultrasonic scanning method is highly dependent on the testing equipment to realize the scanning function. Li et al. [9] proposed an ultrasonic guided waves test method for concrete beams, and successfully tested and evaluated the debonding defects. Wu et al. [10] developed an algorithm based on the smoothed pseudo Wigner-Ville distribution and time-frequency analysis to detect and locate the debonding defect. Mustapha et al. [11,12] studied the sensitivity to debonding defects in sandwich composite structures under different frequency excitation based on ultrasonic guided wave technology. Also, some papers have evaluated interfacial adhesion using the ultrasonic guided waves method. For example, Reis et al. [13] detected the adhesive bond strength of laminated safety glass through energy velocity and attenuation of guided waves. Castaings [14] studied the interfacial adhesive properties of the adhesive between two metallic plates using SH guided waves. Cho et al. [15] characterized interfacial adhesive strength of brazed samples using lamb waves.

In general, the ultrasonic guided wave method has been effective for detecting large defects, but there are few studies on small debonding defects, except for some simulation studies [16]. In the meantime, the nonlinear ultrasonic guided wave method has attracted significant attention because it has the advantage of sensitivity to small defects. Nonlinear ultrasonic guided wave methods can be divided into the nonlinear harmonic and the nonlinear ultrasonic modulation method. For example, Su [17] established a nonlinear ultrasonic testing system for the debonding detection of magnesium alloys, and the results showed that the nonlinear coefficient was increased with the length of the debonding. Yelve et al. [18] found that higher harmonics may be influenced by piezoelectric wafer debonding which leads to incorrect results in damage detection using nonlinear harmonic techniques. Guha et al. [19] analyzed the effect of different frequency on nonlinearity, which was caused by the debonding between transducers and the aluminum plate. Scarselli et al. [20] evaluated the quality of adhesion using nonlinear elastic wave spectroscopy, and nonlinear metrics was used to quantize the nonlinearity based on higher order harmonics. Ciampa et al. [21] developed a nonlinear detection method based on bispectral analysis which could evaluate the cracks and delaminations. However, the detection result of nonlinear harmonic method is also affected by the instruments’ nonlinearity, which means the results may not exactly reflect the state of the defects (contact nonlinearity). Unlike the nonlinear harmonic method, the detection result of the nonlinear ultrasonic modulation method is not affected by the instruments’ nonlinearity [22,23]. For instance, Liu et al. [24] proposed a laser nonlinear ultrasonic modulation testing system and successfully detected the debonding damage in a wind turbine blade. Mandal et al. [25] analyzed the effect of different debonding lengths on a stiffened aluminum through nonlinear wave modulation spectroscopy method. It was found that the relative amplitude of the side-bands with respect to the carrier frequency amplitude is directly proportional to the debonding lengths. The debonding size in the above literature exceeds 200 mm, and in most related research work, it was more than 10 mm [9,26].

Additionally, a considerable amount of research has studied the detection of crack defects, which are usually small, using the nonlinear ultrasonic modulation method, and it has been proved to be effective. For example, Parsons et al. [27] studied the detection effect of nonlinear ultrasonic modulation method with low-frequency excitation. Goursole et al. [28] combined nonlinear ultrasonic modulation and time reversal methods to detect metal crack defects. Ryles et al. [29] detected small fatigue cracks using nonlinear ultrasonic modulation and lamb waves. Hu et al. [30] studied the instantaneous characteristics of nonlinear ultrasonic modulation for metallic structures. Despite those developments in the nonlinear ultrasonic modulation testing method, there is still little research on debonding detection in glass materials, especially for small size debonding defects.

Piezoceramics, especially the lead zirconate titanate (PZT) have been widely used as the transducer in ultrasonic testing technology because of their excellent actuation [31,32,33,34] and sensing [35,36,37,38] performance, and high bandwidth [39,40]. Nieuwenhuis et al. [41] investigated the operability of PZT as the excitation and receive transducers through finite element simulation and a set of experiments. Rajagopalan et al. [42] detected and located the damage of isotropic structures using a PZT array which could excite and receive signals. Ruan et al. [43] successfully detected the damage on wind turbine blades using piezoceramic transducers. Dziendzikowski et al. [44] realized structural health monitoring of composites using a set of PZT transducers and compared the test performance of surface mounted and embedded transducers. Zhu et al. [45] achieved rapid detection and location determination of pipeline leakage using PZT sensors as the transducer. PZT wafers still maintain good performance in glass material [46,47]. Therefore, PZT wafers were used as the actuation and sensing transducer in this paper.

In hidden frame supported glass curtain walls, the debonding defect is the main failure mode, often causing the glass in the curtain wall to fall off. It is necessary to develop an effective testing method for the debonding detection of the hidden frame supported glass curtain wall. Ultrasonic modulation detection method achieves damage detection by exciting two waves with different frequencies. The component with the new frequency is produced by two different frequency waves interacting with the damage, which means the detection result is mainly related to the contact nonlinearity caused by the damage. At present, the debonding size investigated in most of the related research is large, and there is little research on debonding detection for glass materials using nonlinear ultrasonic modulation method [13,14,15,16,17,18]. In this paper, the nonlinear ultrasonic modulation testing method is used to detect small debonding defects in hidden frame supported glass curtain walls. This paper is organized as follows. First, it introduces the testing principle of the nonlinear ultrasonic modulation and the feature extraction method. Second, it describes the experimental setup. Then, the experimental results are presented, and the feasibility of the nonlinear ultrasonic modulation is demonstrated. Finally, the paper concludes with a brief summary.

## 2. Mechanism and Methodology

### 2.1. Theoretical Fundamental of the Nonlinear Ultrasonic Modulation

**Figure 1:**
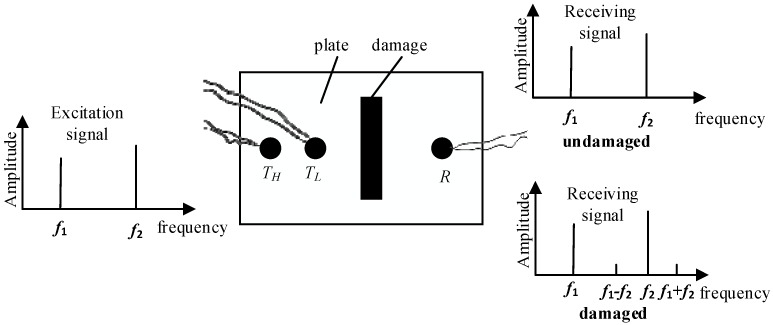
Schematic illustration of nonlinear modulation testing method.

When ultrasonic waves propagate in a nonlinear medium, there will be distortion and deformation to the ultrasonic waves. When a single wave is propagated in the nonlinear medium, there will be harmonics. When multiple waves are propagated in the nonlinear medium, there will be modulation between different frequencies. The ultrasonic modulation detection method achieves damage detection using the modulation phenomena between two excited waves and the medium. If there is a defect in the medium and two waves get together at the defect, there will be new frequency components. The schematic illustration is shown in Figure 1. *T_H_* and *T_L_* are ultrasonic transducers that excite an ultrasonic wave with different frequencies. *R* is the reception transducer. $f_{1}$ and $f_{2}$ are the frequency of the excited two ultrasonic waves, respectively.

In nonlinear acoustics, the relationship between stress and strain can be described by Hook’s law. On the plate, the one-dimensional nonlinear elastic wave equation can be described as [48]:

(1) $$\frac{\partial^{2}u}{\partial t^{2}}-c^{2}\frac{\partial^{2}u}{\partial x^{2}}=c^{2}\beta \frac{\partial u}{\partial x}\frac{\partial^{2}u}{\partial x^{2}},$$

where u is the vibration displacement of particle, x is the distance of wave propagation, c is wave velocity, β is the second-order nonlinear coefficient.

According to wave theory, the solution of the nonlinear Equation (1) can be written as:

(2) $$u(x,t)=u^{\left(0\right)}+\beta u^{\left(1\right)},$$

where $u^{\left(1\right)}$ is the displacement caused by nonlinear fluctuations. Assuming the nonlinear displacement is proportional to the propagation distance, $u^{\left(1\right)}$ can be written as:

(3) $$u^{\left(1\right)}=xh(\tau ),$$

where τ=t−x/c, h(τ) is an unknown function. 

When two waves with different frequency are excitation signals and propagate through the plate, as shown in Equation (4),

(4) $$u^{\left(0\right)}(x,t)=A_{01}\cos(\omega_{1}t)+A_{02}\cos(\omega_{2}t),$$

Then, substituting Equation (4) into Equation (3) results in

(5) $$\begin{array}{ll} u(x,t) & =u^{\left(0\right)}+\beta u^{\left(1\right)}\\ & =A_{01}\cos\omega_{1}t+A_{02}\cos\omega_{2}t+x\beta \{-\frac{A_{01}{}^{2}k_{1}^{2}}{8}\cos(2\omega_{1})t\\ & -\frac{A_{02}{}^{2}k_{2}^{2}}{8}\cos(2\omega_{2})t+\frac{A_{01}A_{02}k_{1}k_{2}}{4}[\cos(\omega_{1}-\omega_{2})t-\cos(\omega_{1}+\omega_{2})t]\} \end{array},$$

where $\omega_{1}$ and $\omega_{2}$ are the frequency of the excitation signals, $k_{1}$ and $k_{2}$ are the wavenumbers of the excitation signals. 

This is abbreviated as:

(6) $$\begin{array}{ll} u(x,t)= & A_{01}\cos\omega_{1}t+A_{02}\cos\omega_{2}t+A_{11}\cos(2\omega_{1})t+A_{12}\cos(2\omega_{2})t\\ & +A_{-}\cos(\omega_{1}-\omega_{2})t-A_{+}\cos(\omega_{1}+\omega_{2})t \end{array},$$

where $A_{11}=-x\beta \frac{A_{01}{}^{2}k_{1}^{2}}{8}$, $A_{12}=-x\beta \frac{A_{02}{}^{2}k_{2}^{2}}{8}$, $A_{-}=x\beta \frac{A_{01}A_{02}k_{1}k_{2}}{4}$, $A_{+}=x\beta \frac{A_{01}A_{02}k_{1}k_{2}}{4}$.

In the frequency domain, from Equation (6), the response function includes not only the fundamental frequency $\omega_{1}$ and $\omega_{2}$, but also the harmonics frequency $2\omega_{1}$ and $2\omega_{2}$, and the side-lobe frequency $\omega_{1}-\omega_{2}$ and $\omega_{1}+\omega_{2}$.

From Equation (6), the nonlinear coefficient can be expressed as:

(7) $$\left\{ \begin{array}{l} \beta_{-}=\frac{A_{-}}{A_{01}A_{02}}\\ \beta_{+}=\frac{A_{+}}{A_{01}A_{02}} \end{array}\right.,$$

where $\beta_{-}$ is the nonlinear coefficient of difference frequency and $\beta_{+}$ is the nonlinear coefficient of the sum frequency.

In the damage detection of the hidden frame supported glass curtain wall, the amplitude of the side-lobe components increases with the nonlinear coefficient. Therefore, nonlinear coefficients can be used to evaluate the damage of the hidden frame supported glass curtain wall.

### 2.2. Empirical Mode Decomposition (EMD)

Empirical mode decomposition (EMD) is an adaptive signal decomposition method, which is widely used in non-stationary signal processing. EMD decomposes the signal according to its own time-scale features without any preset basis functions. It decomposes complex signals into Intrinsic Mode Function (IMF) which includes the local characteristic of different time scales. The essence of EMD is to stabilize the original non-stationary signal [49].

Any signal can be decomposed into the sum of finite IMF by EMD. IMF must satisfy the following two conditions. First, the difference between the number of extrema and zero crossings is less than or equal to 1. Second, the mean value of the envelope determined by local maxima and local minima must be equal to zero. The IMF meeting the above two conditions can be considered as a single component and stable signal [50]. For a non-stationary multi-component signal, the specific process of decomposing it into a series of IMF is as follows.

Step 1: For a given signal x(t), the local maxima and local minima are computed and upper and lower envelop are obtained by connected them using a cubic spline respectively.

Step 2: The mean of the upper and lower envelop $m_{1}(t)$ is calculated and is subtracted from the original signal x(t).

(8) $$h_{1}(t)=x(t)-m_{1}(t),$$

If $h_{1}(t)$ satisfies the conditions of the IMF definition, then $h_{1}(t)$ is the first IMF component, and let $h_{1}=h_{1}(t)$. Otherwise, x(t) is replaced by $h_{1}(t)$ and the previous process is repeated *k* times until the IMF conditions are met and let $h_{1}=h_{1,k}(t)$.

(9) $$h_{1}{}_{,j+1}(t)=h_{1}{}_{,j}(t)-m_{1}{}_{,j+1}(t),j=1,2,\ldots ,k,$$

Step 3: After the first IMF is found, the residue is calculated by subtracted $h_{1}$ from the x(t).

(10) $$r_{1}=x(t)-h_{1},$$

Step 4: Take $r_{1}$ as the new data, repeat step 1, step 2 and step 3, and get the *n*th IMF component $h_{n}$ and *n*th residue $r_{n}$, until the residue $r_{n}$ become too small or $r_{n}(t)$, a monotonic function.

Step 5: The original signal x(t) can be expressed as:

(11) $$x(t)=\sum_{i=1}^{n}h_{i}+r_{n},$$

### 2.3. Discrete Fourier Transform (DFT)

Nonlinear coefficients can reflect the nonlinearity of the material, thus reflecting the damage severity. In the debonding detection in the hidden frame supported glass curtain wall using nonlinear ultrasonic modulation, the received signal contains excitation frequency, harmonic and side-lobe frequency. Therefore, Discrete Fourier Transform (DFT) was used to convert received time-domain signals into frequency-domain signals.

For a limited length series *x*(*n*), 0 ≤ *n* ≤ *N* − 1, DFT is defined as:

(12) $$X(k)=\mathrm{DFT}[x(n)]=\{\begin{array}{cc} \sum_{n=0}^{N-1}x(n)W_{N}^{kn}, & 0\leq k\leq N-1\\ 0, & else \end{array},$$

where $W_{N}=e^{-i(2\pi /N)}$, *n* and *k* are discrete variable. It can be seen that the result after DFT is still a limited length series.

In Equation (12), X(k) is a complex number. The amplitude spectrum was used to extract nonlinear components.

(13) $$A_{k}=A(k)=\left|X(k)\right|,$$

The amplitude integral of frequency band was used to characterize the amplitude feature.

(14) $$A_{i}=\int_{k_{1}}^{k_{2}}A(k)dk,$$

### 2.4. Debonding Evaluation Using Nonlinear Ultrasonic Modulation Method

**Figure 2:**
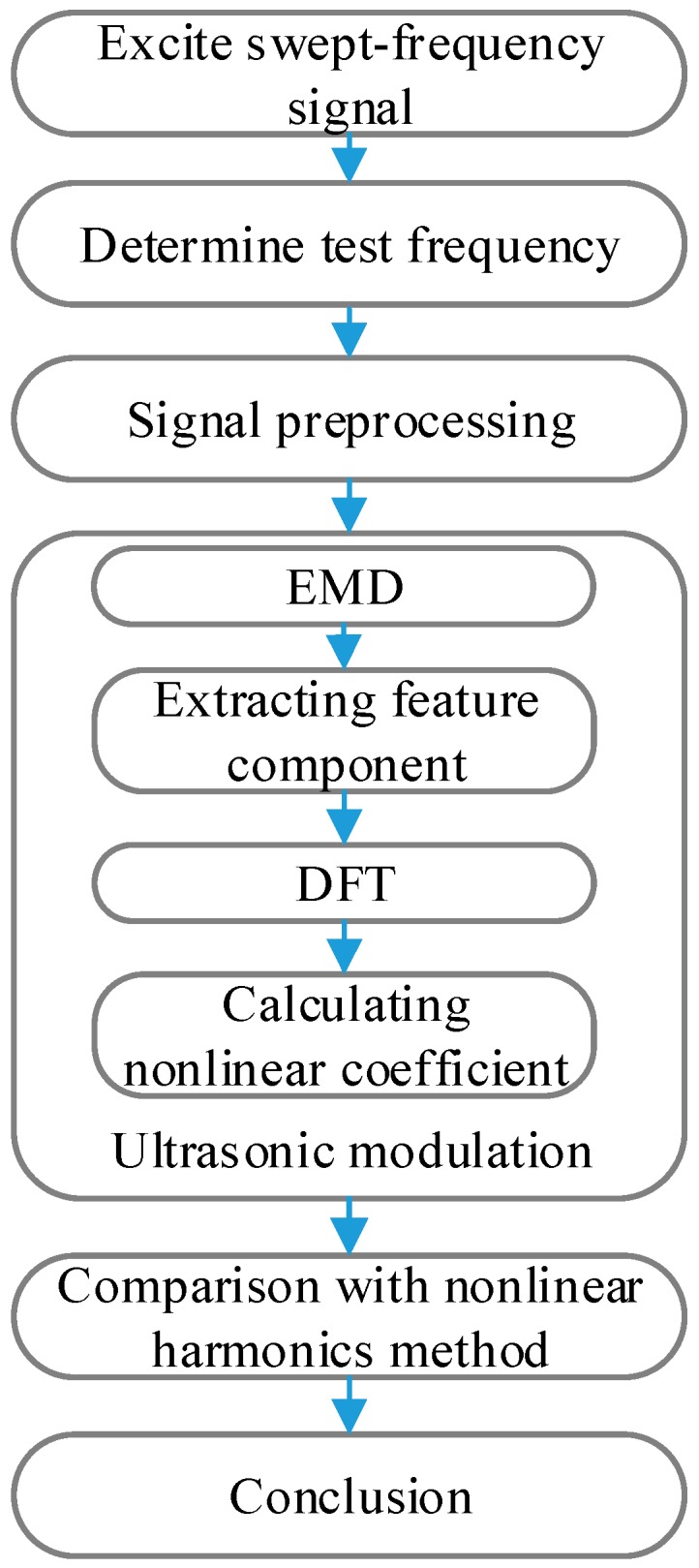
The debonding evaluation process of the hidden frame supported glass curtain wall.

Generally, there is a lot of noise in the received signal, therefore signal preprocessing such as high-pass filtering is needed. The debonding evaluation process of the hidden frame supported glass curtain wall is shown in Figure 2.

Step 1 The sweep experiment of the hidden frame supported glass curtain wall was carried out. Then the frequency response characteristics was obtained.
Step 2 The test frequency of the ultrasonic modulation experiment was determined according to the frequency response characteristics.
Step 3 The experimental platform and test was established using ultrasonic modulation and nonlinear harmonic methods.
Step 4 The receive signal was collected 10 times to reduce the experimental error, and then put through the high-pass filter to reduce the noise.
Step 5 The IMF components were obtained after EMD. According to the IMF energy, the IMF component containing the nonlinear damage information was selected as the characteristics component.
Step 6 The nonlinear coefficient of hidden frame supported glass curtain wall was calculated based on the DFT.
Step 7 The results were compared with the ultrasonic nonlinear harmonics method to verify the feasibility of the method.

## 3. Experimental Setup

**Figure 3:**
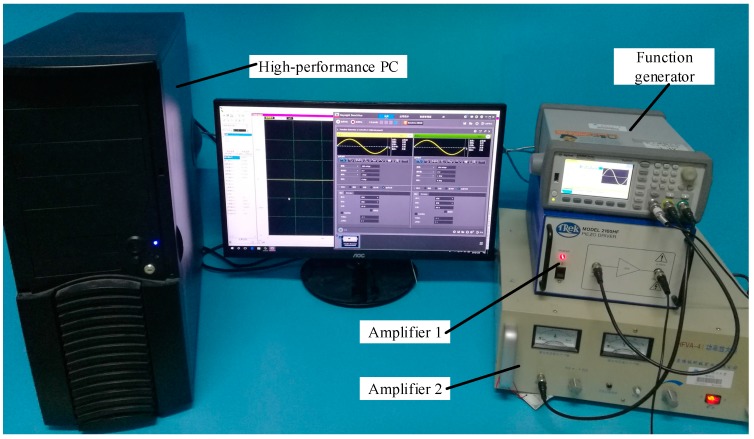
Experimental setup.

The nonlinear ultrasonic modulation testing system was established based on the theoretical research. The experimental system consisted of an Agilent 33522B function generator (Agilent Technologies, Ltd., Palo Alto, CA, USA), TREK2100HF amplifier (Trek, Inc., New York, NY, USA), HFVA-41 amplifier (Nanjing Buddha Science and Technology Industry Co., Ltd., Nanjing, China), and a PCI-20614 data acquisition card (Sichuan Tupu TT & C Technology Co., Ltd., Chengdu, China) embedded in the host, the high-performance PC. The physical diagram of the experimental system is shown in Figure 3.

**Figure 4:**
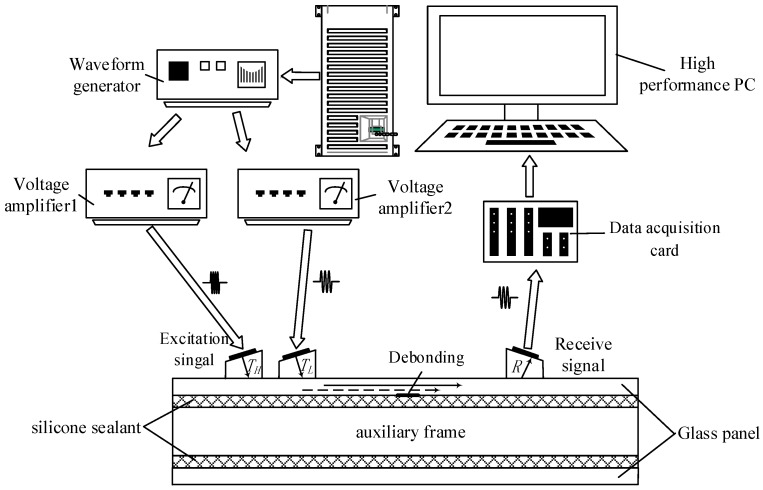
The experimental block diagram of the nonlinear ultrasonic modulation testing method.

During the experiment, two sine waves with different frequency were excited at the same time by the function generator. The generated two ultrasonic waves were amplified and were obtained through amplifier 1 and amplifier 2. Then, the amplified signals drive the transducer *T_H_* and *T_L_* to produce a sufficiently strong ultrasonic wave. The generated two ultrasonic waves propagate in the medium, and if there is a defect, the nonlinear modulation phenomenon appears when two waves meet at the defect. The transducer *R* receives the signal and it is transmitted to the high-performance PC by a data acquisition card. The detailed experimental block diagram is shown in Figure 4.

**Figure 5:**
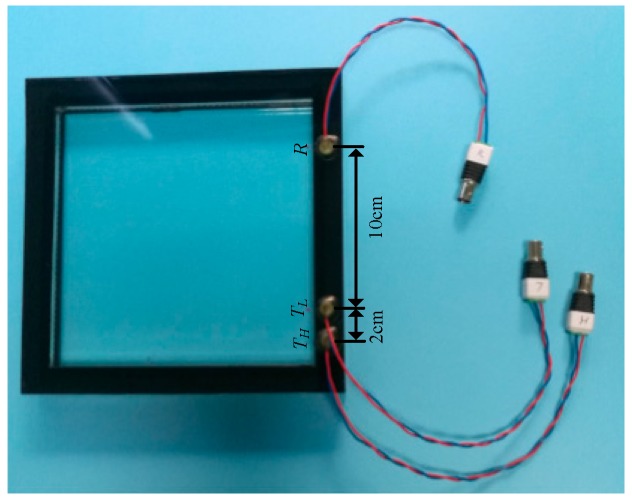
Hidden frame supported glass curtain wall with PZTs.

The experimental object is a hidden frame supported glass curtain wall measuring 200 mm × 200 mm × 24 mm. It is mainly composed of two glass plates and an adhesive structure. The two glass plates are glued together with a structural silicone sealant. Three PZTs were bonded on the side of the glass plate to excite and receive the signals. The sample of the hidden frame supported glass curtain wall with PZTs is shown in Figure 5. It should be noted that three PZTs were distributed on the same line. *T_H_* and *T_L_* are excitation transducers, and *R* is the reception transducer. The resonant frequency of the PZTs is 2 MHz. The diameter is 8 mm and the thickness is 1 mm. The PZT transducers were bonded to the glass plate using Leaftop 9903 AB glue. Leaftop 9903 AB glue has high shear and impact resistance which ensures good bonding between the PZT transducers and the glass plate. The distance between *T_H_* and *T_L_* is 20 mm, and the distance between *T_L_* and *R* is 100 mm.

## 4. Experiments and Analyses

### 4.1. Experimental Process

**Figure 6:**
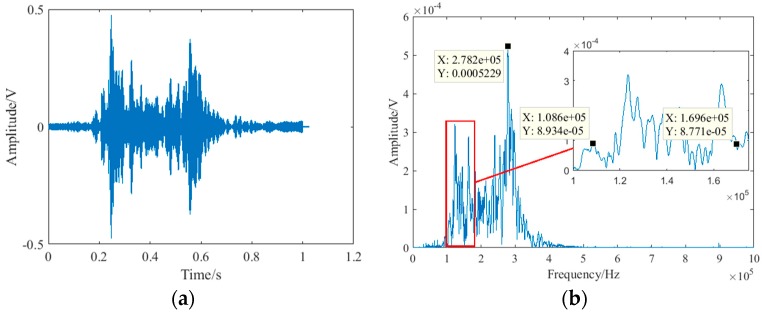
The received sweep signal: (**a**) time-domain; (**b**) frequency-domain.

The nonlinear ultrasonic modulation method is sensitive to contact nonlinearity which is mainly caused by damage in material. However, the nonlinear effect cannot be completely zero for undamaged material. Even for undamaged material, there will inevitably be a small amount of contact nonlinearity. On the other hand, small debonding defects are the main research object. Therefore, to obtain a stronger nonlinear effect and better detection results, a sweep experiment was carried out to obtain a stronger response. The range of the sweep signal is from 0 kHz to 500 kHz. The sweep signal lasts for 1 s to make sure the response is sufficient, so that the obtained glass curtain wall frequency response will be more accurate. Then, the transducers *T_H_* and *T_L_* are excited at the same time, and received by transducer *R*. The sweep signal was a sine wave with an amplitude of 3 V. The sweep results are shown in Figure 6.

**Figure 7:**
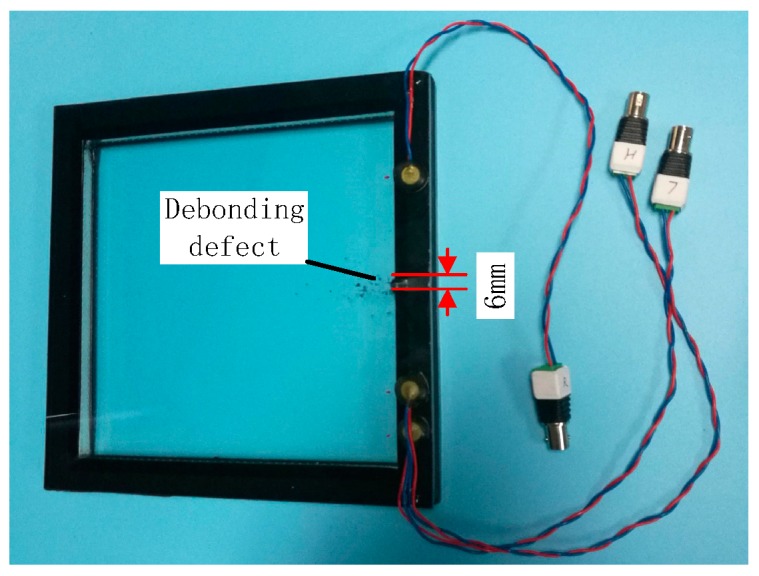
Artificial defect with 6 mm debonding.

From Figure 6, the frequency response was more obvious from 100 kHz to 350 kHz, and the amplitude of frequency response is the strongest at 278.2 kHz. The side-lobe frequency contains sum frequency and difference frequency which can both be used to calculate the nonlinear coefficients by Equation (7). Therefore, according to the sweep result, the frequencies of the excitation signal were 108.6 kHz and 169.6 kHz, and the sum frequency was 278.2 kHz to acquire a strong response. The amplitude of both the two excited sine signals was 3 V and lasted for 0.1 s. After the amplifier, the signal was amplified 50 times to drive the transducer *T_H_* and *T_L_*. The sampling rate of the data acquisition card is 2 MHz. The debonding defects were made by separating the glass plate from the structural silicone sealant with a slender iron sheet. The slender iron sheet was carefully inserted into the contact interface between the silicone structural adhesive and the glass plate, and then carefully pulled out. Because the silicone structural adhesive is elastic, it will almost cling to the glass plate when the slender iron sheet is pulled out. The thickness of the debonding defect can be ignored. Once a group of experiments were completed, the length of the debonding was increased and the next group of experiments were carried out. Five debonding states (0 mm, 3 mm, 6 mm, 9 mm, and 12 mm) were investigated. Figure 7 shows the artificial defect with a 6 mm debonding. Although the transducer *T_H_* and *T_L_* was close to the boundary, the nonlinear ultrasonic modulation method was mainly analyzed in frequency-domain. Therefore, if the signal contains the sum frequency information, the reflected wave also contains the sum frequency information, which has little effect on the result of the frequency domain. In data acquisition, the reception signal was collected 10 times to reduce random errors and increase the reliability of the measurement.

**Figure 8:**
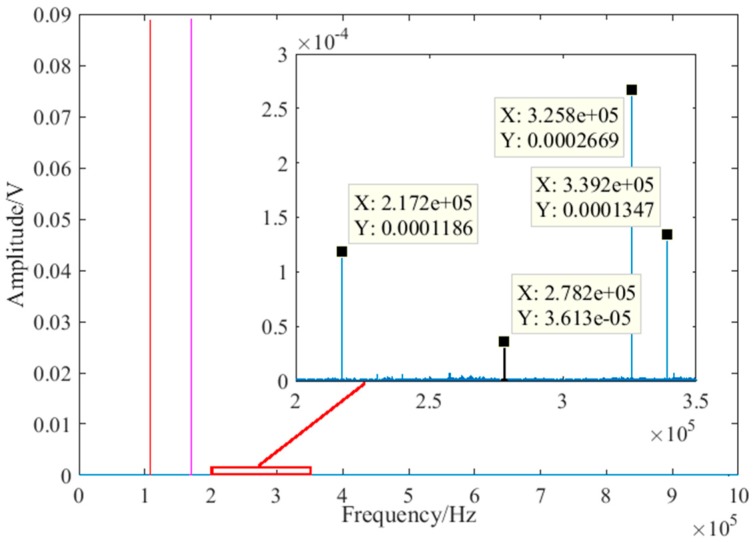
The received modulation signal under undamaged conditions.

The received modulation signal under undamaged conditions is shown in Figure 8. The amplitude of the side-lobe is very small compared with the fundamental signal. From the enlarged detail, it can be seen that the received signal contains the fundamental frequency, harmonics, and side-lobe. The amplitude of the harmonics was generally larger than the side-lobe, because the nonlinear harmonics not only include the contact nonlinearity but also the instrument nonlinearity. In addition, since the frequency response is the strongest at the sum frequency, the amplitude of sum frequency is larger than the amplitude of difference frequency.

### 4.2. Debonding Damage Evaluation Using Nonlinear Ultrasonic Modulation

**Figure 9:**
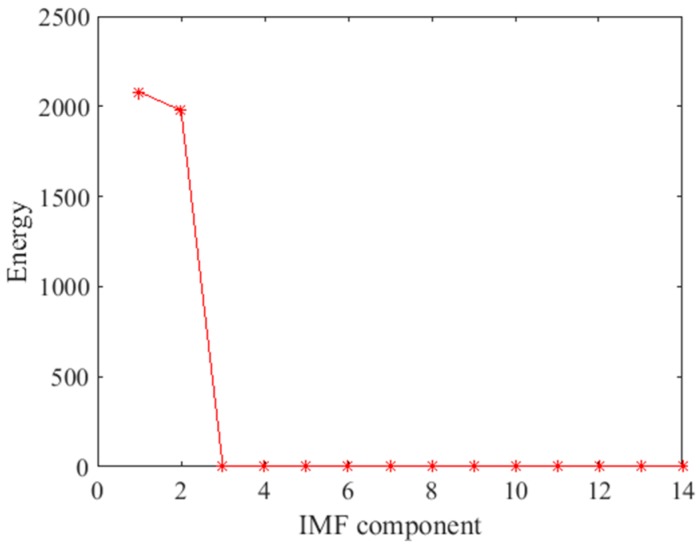
The energy of each IMF component and residue.

**Figure 10:**
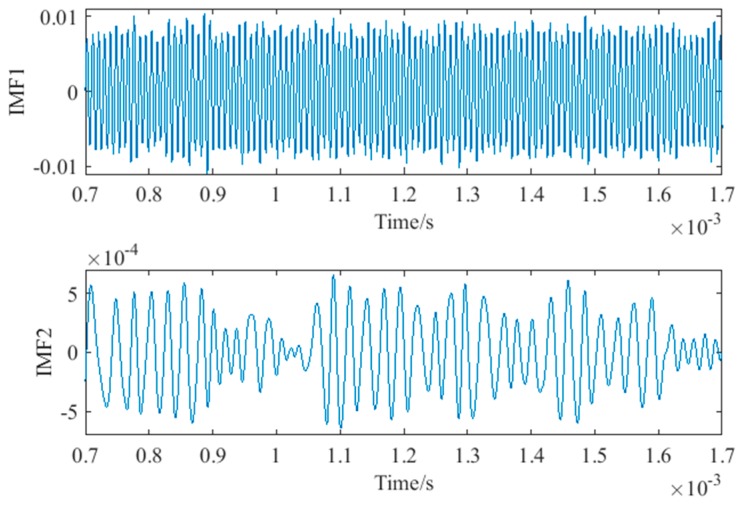
The local details of IMF1 and IMF2 components of the no damage signal after EMD.

In the preprocessing, a high-pass filter was used to remove noise from the collected signals. The cutoff frequency of the high-pass filter was 50 kHz, and the gain of the high-pass filter was 1. Then, the signals were decomposed into IMF components of different time scale and residue by EMD. In total, 13 IMF components and residue were obtained. The energy of each IMF component was calculated, and this is shown in Figure 9. It was found that the energy is concentrated in IMF1 and IMF2. The energy of IMF1 and IMF2 accounts for 99.89% of the total energy. In other words, IMF1 and IMF2 contain the main feature information. Therefore, IMF1 and IMF2 were used to reconstruct the signal. The decomposed IMF1 and IMF2 components of the no damage signal are shown in Figure 10. Taking into account the large amount of data, the IMF1 and IMF2 were locally amplified.

**Figure 11:**
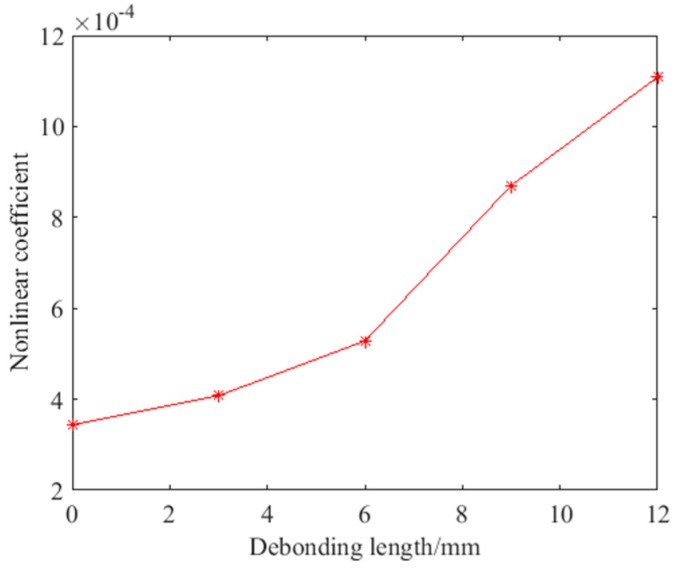
The variation curves of the nonlinear coefficient under different debonding states.

Then, the DFT was used to get the frequency-domain signal of the reconstructed signal, and the nonlinear coefficients under different debonding states were calculated using Equations (7) and (14). The variation curve of the nonlinear coefficient is shown in Figure 11. It can be seen that even if there is no damage, there will still be less nonlinearity, but the nonlinear coefficient is small. When a debonding defect occurs, the nonlinear coefficient increases gradually, but the growth rate of the nonlinear coefficient is different with the increase in the size of the debonding defect. When the debonding defect is small, the growth rate is small, for example, when the debonding length is 3 mm the minimum growth rate is 18.64%. When the debonding defect is large, the growth rate is also large, for example, when the debonding length is 9 mm, the growth rate reaches the maximum of 64.77%. When the debonding defect is 6 mm and 12 mm, the growth rate is more than 25%. The difference in the nonlinear coefficient between a 3 mm debonding defect and no damage is very small. Debonding detection with a small defect size is a challenge, and the accuracy of debonding defect evaluation decreases with the decrease in the debonding size. In addition, the nonlinear coefficient reflects the nonlinear effect that exists in the material. The ultrasonic modulation detection method is mainly sensitive to the contact nonlinearity which is related to the debonding states. However, in the actual detection, material with no damage also has weak contact nonlinearity. Therefore, it is necessary to set a baseline for the nonlinear coefficient. When the nonlinear coefficient exceeds the baseline, it is considered that there is a debonding defect, otherwise it is an unavoidable contact nonlinearity in the material. Figure 11 reflects the relationship between the nonlinear coefficient and the debonding state, and it is clear that the nonlinear coefficient increases with the debonding length. The nonlinear ultrasonic modulation method can be used to detect debonding defects in the hidden frame supported glass curtain wall.

**Figure 12:**
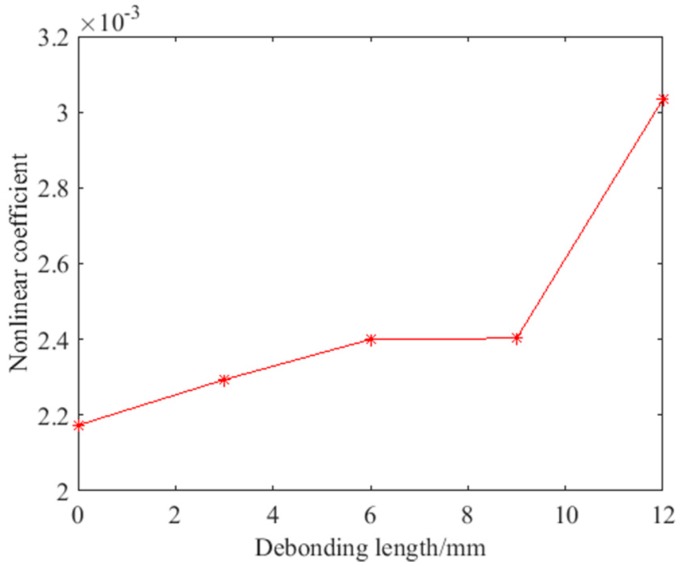
The variation curves of the nonlinear coefficient using nonlinear harmonics method.

The ultrasonic nonlinear harmonics method was also applied to detect the debonding defect and compared with the nonlinear ultrasonic modulation. The frequencies of the excitation signal were 139.1 kHz and the frequency of the second harmonic was 278.2 kHz to acquire a strong response. The signal processing of the nonlinear harmonics method was the same as the nonlinear ultrasonic modulation method. The received signal was denoised by a high-pass filter, then decomposed into IMF components by EMD, and the nonlinear coefficient was calculated after DFT. The variation curves of the nonlinear coefficient using the nonlinear harmonics method are shown in Figure 12. The detection result of the nonlinear harmonic method includes not only contact nonlinearity but also the instrument nonlinearity. Therefore, the nonlinear coefficient of the nonlinear harmonics method was larger than that of the nonlinear ultrasonic modulation method. Similar to the nonlinear modulation method, the nonlinear coefficient increases with the debonding length. The growth is obvious except when the debonding length is 9 mm. Because the amplitude of the nonlinear coefficient is large, the growth rate is relatively small. The growth rate exceeds 25% only when the defect is 12 mm. The nonlinear coefficient increased with the debonding length. In other words, the nonlinear harmonics method can still detect debonding defects in hidden frame supported glass curtain walls.

To further compare the nonlinear ultrasonic method and nonlinear harmonics, normalization was performed, and the mean squared error (MSE) was used to evaluate the result. The MSE of the two methods are shown in Table 1. The MSE of both methods was small. It also proves the feasibility of both methods for debonding detection. The MSE of nonlinear ultrasonic modulation are smaller than that of nonlinear harmonics. The MSE of the nonlinear ultrasonic modulation method has been improved by 41%, thus, the nonlinear ultrasonic modulation method is more accurate than the nonlinear harmonics method.

### 4.3. Debonding Detection Under Different Temperatures

**Figure 13:**
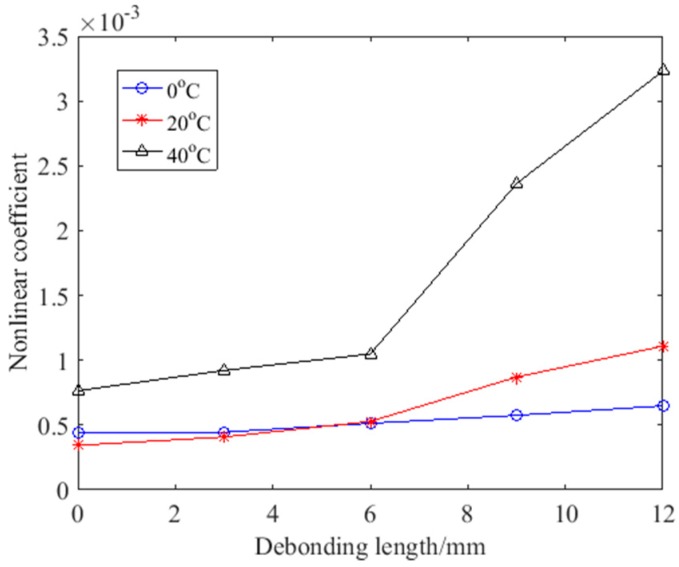
The variation curves of the nonlinear coefficient under different temperatures.

The working environment of the hidden frame supported glass curtain wall varies, in particular, the temperature varies greatly in different places and time. Therefore, detection using nonlinear ultrasonic modulation at different temperatures was studied. The low and high temperature environment were controlled by refrigerator and thermal excitation devices. An infrared thermometer was used to verify temperature. The thermal excitation device was independently developed by the authors’ [51]. The model of the refrigerator is Haier BC/BD-202HT (Haier, Qingdao, China). The model of the infrared thermometer is Victor 307C (Xian Beicheng electronic limited liability company, Xian, China). The variation curves of the nonlinear coefficient under different temperatures are shown in Figure 13. The detection results of the test at different temperatures of the same debonding state were different. In a high temperature state, the nonlinear coefficient values were generally higher which caused larger growth with the increase in debonding length. The growth rate is generally over 13%, for example, when the debonding length is 9 mm, the growth rate reaches a maximum of 125.39%. When the temperature is low, the growth rate is basically over 12% except when the debonding length is 3 mm, it is only 1.74%. Because of the influence of temperature, the nonlinear coefficient changes, but it still reflects the defects in the material.

**Figure 14:**
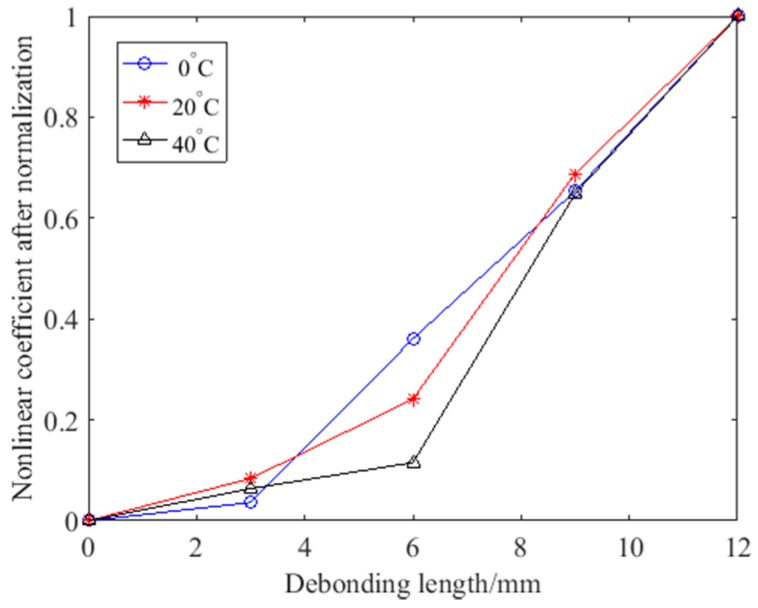
The variation curves of the nonlinear coefficient after normalization under different temperatures.

To compare the differences at different temperatures, normalization was carried out. After normalization, the change trend of the nonlinear coefficient at different temperatures will more obvious, and this is shown in Figure 14. It can be seen that the change trend for different temperatures was similar. The nonlinear coefficient increases slowly when the debonding length is small and increases rapidly when the debonding length is larger at different temperatures. The growth rate of the normalized nonlinear coefficient was closer at different temperatures, which means the nonlinear coefficient still reflects the debonding defect at different temperatures. Although the temperature has an impact on the test results, debonding detection at different temperatures is still reliable.

### 4.4. Debonding Detection Under Different Impact

**Figure 15:**
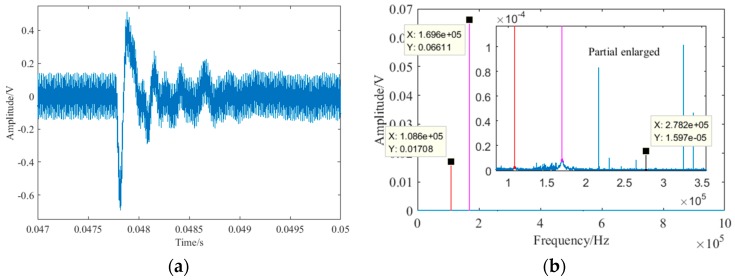
The received impact signal of 500 mm: (**a**) local details of impact response in the time-domain; (**b**) impact signal in the frequency-domain.

**Figure 16:**
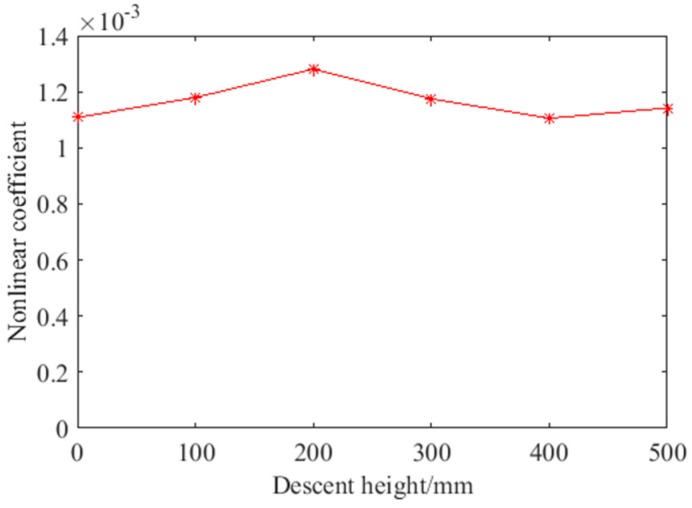
The received impact signal at different descent heights.

As an external enclosure and decorative structure of high-rise buildings, the vibration caused by wind load is also an important factor which may affect the detection result. The nonlinear ultrasonic modulation method completed defect detection in the frequency-domain. The impact response contains a very wide range of frequency components. The spectrum of the impact response was similar. Therefore, to further verify the anti-interference of vibration using the nonlinear ultrasonic modulation method, the detection effect under different single impacts were studied. The impact experiment was carried out using a small plastic ball with a mass of 2.05 g when the debonding length was 12 mm. In the experiment, the ball knocked the glass curtain wall at different heights to simulate various impacts. The impact location was between the excitation and the receive transducer, directly above the debonding defect to maximize the interference. The descent heights of the ball were 100 mm, 200 mm, 300 mm, 400 mm and 500 mm respectively. The received impact signal of 500 mm was shown in Figure 15. Because of the large amount of data, the local details of the impact response in the time-domain are shown in Figure 15a. In the time-domain, the maximum response of the ball impact was obviously greater than the sine signal of the excitation. However, in the frequency-domain, there was no obvious changes. This is because the energy of the impact in the frequency-domain was small and dispersive compared with the excitation frequency and the sum frequency. After the calculation, the received impact signal at different descent height is shown in Figure 16. It can be seen that the nonlinear coefficient with or without ball impact is very close. According to Equation (7), the nonlinear coefficient is mainly related to the amplitude of two excitation frequencies and sum frequency. Therefore, as long as the frequency of the vibration interference is not the same as the excitation frequency and the sum frequency, the vibration interference has little influence on the detection result. The nonlinear ultrasonic modulation method has anti-interference properties for vibration to a certain extent.

## 5. Conclusions

In this paper, the nonlinear ultrasonic modulation method was developed for debonding detection in hidden frame supported glass curtain walls. A series of experiments were carried out to verify the feasibility and effectiveness of the nonlinear ultrasonic modulation method. First, a nonlinear ultrasonic modulation testing system was established. The sweep signal experiment was performed to guide the determination of the excitation frequency and obtain the strongest modulation signal. The detection results from the nonlinear ultrasonic modulation method showed that the nonlinear coefficient increases with the debonding length. The nonlinear ultrasonic modulation method can successfully detect debonding defects of hidden frame supported glass curtain walls. Furthermore, the nonlinear harmonics method was examined and compared with the nonlinear ultrasonic modulation method. Normalization and MSE were adopted to compare the two methods. The MSE of the nonlinear ultrasonic modulation method was improved by 41% compared with the nonlinear harmonics method. In addition, to prove the anti-interference performance of the nonlinear ultrasonic modulation method, the detection effect at different temperatures and impact was studied. The results showed that although the nonlinear coefficient values were different at different temperatures, the change trend is consistent with the debonding length at different temperatures. The interference of impact vibration does not significantly affect the detection result.

In summary, the nonlinear ultrasonic modulation method can be used to detect debonding defects in hidden frame supported glass curtain walls. The detection of debonding defects can be realized at different temperatures. The results of this method include only contact nonlinearity, and the results were more accurate. The method lays a foundation for debonding detection in glass curtain walls using nonlinear ultrasonic testing method. However, the detection accuracy of debonding defects could be further improved. The next step is to study the localization and imaging of the debonding defect in glass curtain walls.

## Figures and Tables

**Table 1:** MSE of nonlinear ultrasonic modulation and nonlinear harmonics.

Detection Method	MSE
Nonlinear ultrasonic modulation	0.0974
Nonlinear harmonics	0.1660
